# 25-Hydroxyvitamin D variability within-person due to diurnal rhythm and illness: a case report

**DOI:** 10.1186/s13256-018-1948-9

**Published:** 2019-02-04

**Authors:** Christine B. French, Sharon L. McDonnell, Reinhold Vieth

**Affiliations:** 1 GrassrootsHealth, 315 S. Coast Hwy 101, Suite U-87, Encinitas, CA 92024 USA; 2 Department of Nutritional Sciences, and Department of Laboratory Medicine and Pathobiology, University of Toronto, 27 King’s College Circle, Toronto, Ontario M5S 1A1 Canada

**Keywords:** 25-hydroxyvitamin D, Diurnal variation, Trough sampling

## Abstract

**Background:**

Vitamin D nutrition research requires accurate measures of circulating 25-hydroxyvitamin D. Our objectives were to test whether a diurnal fluctuation in blood-spot concentrations of 25-hydroxyvitamin D can be demonstrated statistically in a single individual, and whether such fluctuation is affected by the pre-dose versus post-dose timing of the blood draw.

**Case presentation:**

The participant in this case study was a generally healthy Caucasian woman in her 40s who has taken 5000 IU vitamin D3 supplement at midday for over 1 year. Each blood sample was drawn individually from a finger prick onto filter paper at morning, midday, or night, on 4 days (three groups of five individual blood samples per collection day). On days 1 and 2, the midday samples were collected approximately 1 hour after the supplement was taken; on days 3 and 4, the midday samples were collected within an hour prior to supplementation (the classical, daily “trough” value for a drug). There was a significant daily pattern of variation in 25-hydroxyvitamin D concentrations (analysis of variance *p* ≤ 0.02 for 3 of the 4 days): peak midday mean 25-hydroxyvitamin D was approximately 20% higher than in the morning, and approximately 13% higher than in the evening. Trough sampling produced no significant difference in 25-hydroxyvitamin D compared to sampling an hour after the dose. An incidental finding was that acute illness during the study was related to acutely lower 25-hydroxyvitamin D at every sampling time in the day (*p* < 0.00001).

**Conclusions:**

There was a consistent diurnal variation in 25-hydroxyvitamin D, with the peak at midday. There was no difference between trough versus post-dose blood draws. Acute illness may acutely lower serum 25-hydroxyvitamin D levels. Because within-person, within-day variability in 25-hydroxyvitamin D is approximately 20%, sampling time introduces systematic error in vitamin D nutritional assessment that is bigger than random analytical error or choice of assay method.

**Electronic supplementary material:**

The online version of this article (https://doi.org/10.1186/s13256-018-1948-9) contains supplementary material, which is available to authorized users.

## Background

Since vitamin D is generated in the skin when it is exposed to ultraviolet (UV) light in the UVB range, it is logical that the serum 25-hydroxyvitamin D [25(OH)D] concentration – the measure of vitamin D nutritional status – should exhibit a seasonal rhythm in populations that inhabit temperate latitudes [1]. Serum 25(OH)D levels have a long half-life and are sustained by vitamin D and 25(OH)D stored in muscle and adipose tissues [2]. Because serum 25(OH)D exhibits a long apparent circulating half-life, it was long presumed that the time of day for drawing a sample would not matter. After an isolated large dose of vitamin D is consumed, the serum 25(OH)D rises over several days and it stays up for several months [2, 3]. But with ongoing daily doses of vitamin D, it takes months to reach a plateau in serum 25(OH)D [4], and we are not aware of any studies that have investigated whether serum 25(OH)D fluctuates in the hours after each individual dose.

Assay methodologies have received much attention to standardize assessments of vitamin D nutritional status [5, 6]. Remarkably, none of the 25(OH)D assay experts seem to have taken into account the within-participant variability in 25(OH)D measurement. Recent reports have demonstrated convincingly, that for various population groups, there are substantial diurnal rhythms in serum 25(OH)D [7, 8]. Although the Recommended Dietary Allowance (RDA) for vitamin D for adults up to age 70 years is 600 IU/day [9], the Endocrine Society recommends 2000 IU/day [10], and many Americans have chosen to consume higher daily doses of vitamin D. These things have led us to address the question of whether it is possible to detect within-day fluctuations in serum 25(OH)D within a single individual, and whether those fluctuations might be attributable to the ongoing cycles of daily consumption of a physiological amount of vitamin D, 5000 IU/day [1, 11].

## Case presentation

The study was conducted with a single participant of the GrassrootsHealth cohort, a large prospective cohort study in which volunteers submit home blood-spot 25(OH)D test kits and complete an online health questionnaire. The case study participant is a generally healthy Caucasian woman in her mid-40s who resides in Southern California, USA, at latitude 34 degrees north. The individual enrolled in the GrassrootsHealth study in December 2008, has an established history of 25(OH)D measurements above 60 ng/ml (to convert values to nmol/L, multiply by 2.5). She had been taking a daily vitamin D supplement of 5000 IU at midday for over 1 year prior to the present study day 1. No other supplements were taken on a regular basis. The GrassrootsHealth study was approved by the Western Institutional Review Board (Olympia, WA, USA), and the individual whose results are presented signed a consent form to use the data.

Measurement of blood-spot 25(OH)D concentrations for this study was determined by analysis of dried blood-spot test kits using liquid chromatography-mass spectroscopy (LC-MS/MS) by ZRT Laboratory (Beaverton, OR, USA). The 25(OH)D results were calibrated to match the values obtained by serum-based assay methods, and the ZRT assay has been validated against the Vitamin D External Quality Assessment Scheme (DEQAS) LC-MS/MS consensus group (*R*^*2*^ value of 0.998). Analysis of 25(OH)D from dried blood-spot cards using LC-MS/MS has been validated against the radioimmunoassay method (*R*^2^ = 0.91 and a slope not different from 1.0) [12].

Imprecision of the assay method, at a mean 25(OH)D of 50.7 ng/ml (the quality control value closest to the levels reported here) was 7.7% coefficient of variation [13]. To account for analytical variability and to be powered to detect a 5% fluctuation in 25(OH)D over the course of the day, or from 1 week to the next, the test participant provided sets of five independently drawn samples at 8-hour intervals for a total of 15 samples on each of four testing days.

The first set of two testing days, that is sampling day 1 and day 2, were 7 days apart in mid-October 2017. During this time, our participant continued her normal diet, exercise, sleep, and supplementation routines. Apart from supplementation, her normal diet contains little vitamin D, with occasional (less than once a week on average) fish intake and limited dairy (primarily cheese). Typical sun exposure is concentrated in the morning hours with outdoor exercise (run or walk) and light gardening prior to 09:00.

The second set of two testing days, that is day 3 and day 4, were 13 days apart in mid to late-November 2017. For these two collection days, the theoretical “trough” values were obtained, in that the midday sample was taken just prior to the daily dose. Our participant reported the onset of a cold 24 hours after the final sample collection of day 3. There were no changes in supplementation routine, but diet, exercise, and sleep were all adjusted to accommodate the symptoms of the cold. The day 4 collection day was postponed from the originally scheduled 1 week follow-up, until the cold had subsided, 13 days after the day 3 collection date.

Statistical analyses were performed using the R software (www.r-project.org), and confirmed by using SPSS version 2017. Although the blood samples were taken by repeated sampling on one individual, the statistical analysis treated each separately drawn sample as an individual value, not paired with other values. At each time point, five separate blood samples were taken to generate a group of values. This approach was used in order to address the question of whether, in one individual, mean blood 25(OH)D values that were drawn at different times of the day were significantly different. Samples were grouped based on the collection time, and on the timing of the blood draw in relation to the dose (on days 1 and 2, blood was sampled an hour after the dose, and on days 3 and 4, blood was sampled at the theoretical “trough” time for a drug, within an hour before the dose). Comparisons across each day were done by one-way analysis of variance (ANOVA), followed by Bonferroni-corrected comparisons between individual groups of samples, because we were investigating whether average 25(OH)D results for groups of blood-spot samples, collected at morning, midday, and night, from one person would fluctuate over the course of each day.

## Results

**Fig. 1:**
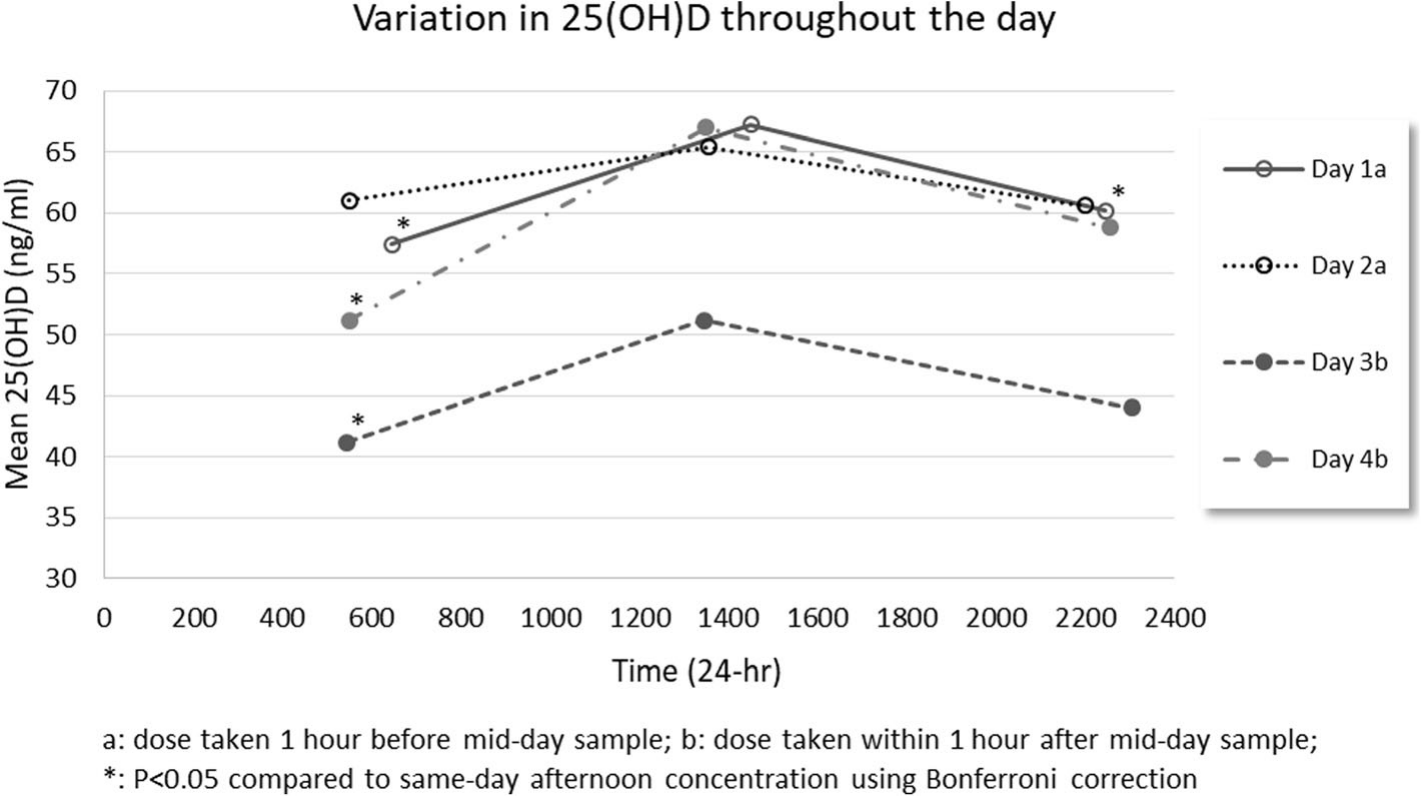
Repeated pattern of daily fluctuation in blood-spot 25-hydroxyvitamin D levels. Each dot represents the mean measurement of five separately sampled blood-spot 25-hydroxyvitamin D test samples taken from one person. At days 1 and 2, the midday samples were taken 1 hour after the preceding daily, 5000 IU dose. At days 3 and 4, the midday samples were theoretical trough values, that is, sample was taken approximately 24 hours after the preceding daily, 5000 IU dose. *25(OH)D* 25-hydroxyvitamin D

On days 1 and 2, the 5000 IU daily dose of supplement was taken approximately 1 hour before the midday sample collection (Table 1). For the day 1 samples, there was a significant fluctuation in 25(OH)D throughout the day, *p* = 0.01, based on one-way ANOVA. For the day 2 samples, the result of one-way ANOVA was *p* = 0.21. There was no significant difference in blood-spot 25(OH)D between the 2 days at any time point. The pre-planned, one-way ANOVA analysis of results pooled from the 2 days in which the 5000 IU dose was taken an hour before the midday sample demonstrated fluctuation in blood-spot 25(OH)D across the day, *p* = 0.002. Figure 1 shows the mean measurement at each time point and Additional file 1 shows the mean measurement along with all five individual sample measurements at each time point. Post hoc testing using Bonferroni correction showed that midday values were significantly higher than morning and evening results for the pooled values and day 1 individually. Mean 25(OH)D concentration at midday was 12% higher (*p* = 0.009) than the mean of the morning samples (17%, *p* = 0.047 for day 1 and 7%, *p* = 0.41 for day 2) and 10% higher (*p* = 0.009) than the mean of the evening samples (12%, *p* = 0.01 for day 1 and 8%, *p* = 0.49 for day 2).

**Table 1:** Dates and times of dried blood-spot sample collections and supplement intake

Day	Date (MM/DD/YY)	Time (24-hour)			
		Morning sample collection	Midday sample collection	Night sample collection	Supplement taken
1	10/11/17	06:45	14:50	22:45	13:40
2	10/18/17	05:50	13:55	22:00	13:10
3	11/15/17	05:45	13:45	23:05	13:50
4	11/28/17	05:50	13:50	22:55	14:40

On days 3 and 4, the supplement was taken within an hour after the midday sample collection. For the day 3 samples, a one-way ANOVA demonstrated fluctuation in blood-spot 25(OH)D across the day (*p* = 0.02). Likewise, for the day 4 samples, a one-way ANOVA demonstrated fluctuation in blood-spot 25(OH)D across the day (*p* = 0.0003). Since there was a significant difference in mean blood-spot 25(OH)D at each of the three time points comparing day 3 versus day 4 (*p* < 0.01 for each), it was not appropriate to conduct a pooled one-way ANOVA analysis for these samples. However, the diurnal pattern for day 3 and day 4 separately was evident. For day 3, the 25(OH)D concentration at midday was 24% higher (*p* = 0.007) than the morning concentration and 16% higher (*p* = 0.21) than the evening concentration. For day 4, the 25(OH)D concentration at midday was 31% higher (*p* = 0.0003) than the morning concentration and 14% higher (*p* = 0.07) than the evening concentration. The midday 25(OH)D values of day 4 taken at the theoretical “trough”, pre-dose sampling time point, were not significantly different from the 1-hour post-dose values of pooled days 1 and 2 (*t*-test *p* = 0.742, *n* = 15, df = 13).

Mean 25(OH)D concentrations on day 3 were approximately 25% lower than the mean values for each of the other 3 days and at all three collection time points (ANOVA *p* < 0.00001, and *p* < 0.013 for all pairwise Bonferroni-corrected comparisons). The lower 25(OH)D values for day 3 are clearly evident in the scattergram of 25(OH)D values (Fig. 1). Just after sampling day 3, our participant reported the onset of a cold; there were no changes in supplementation routine, but diet, exercise, and sleep were all adjusted to accommodate the illness.

## Discussion

This case report demonstrates several features of clinical interest. Firstly, based on pharmacological theory, taking the midday sample before taking the day’s dose should have produced midday trough levels for 25(OH)D because the midday sample was taken at approximately 24 hours after the preceding daily dose (Table 1). However, post hoc testing using Bonferroni correction showed consistently that midday values were significantly higher than morning results (Fig. 1). The blood level of 25(OH)D can be approximately 10 to 20% higher at midday compared to samples drawn in early morning or at night.

Furthermore, this confirms within a single individual, the diurnal variability shown in larger population groups [7, 8]. If 25(OH)D levels are to be compared within one person, or between groups, it can be more important to be consistent with the time of day in which a sample is drawn from a participant, than it is to aim for analytical perfection by the laboratory. Between-run coefficients of variability for 25(OH)D assay methods tend to be in the 5–10% range [5, 6], which is less than the same-day, within-participant variability demonstrated here.

In addition, it is common to want to assess the vitamin D nutritional status of patients taking higher than usual amounts of vitamin D. The previous reports of a diurnal pattern in serum 25(OH)D dealt with populations having low serum 25(OH)D, below 25 ng/mL [7, 8]. Therefore, it was not known whether the diurnal pattern could be seen in someone taking a higher dose of vitamin D. We found a repeated circadian pattern across four different sampling days in a participant with 25(OH)D concentrations consistently over 50 ng/mL. Furthermore, the timing of taking a larger dose of vitamin D in relation to the sample for 25(OH)D assay was found not to make a significant difference – at least not within an hour after the dose.

Lastly, we present a fortuitous observation, that despite continued daily consumption of 5000 IU vitamin D, the blood-spot 25(OH)D level declined by approximately 25% at all three time points on the day immediately before the onset of an illness (day 3, Fig. 1). By the time symptoms subsided, 2 weeks later, the day 4 blood-spot 25(OH)D concentrations had returned to match the pre-illness concentrations of days 1 and 2. In the debates about the relationship between vitamin D status and disease, Autier *et al.* have postulated, controversially, that that low 25(OH)D concentrations are a consequence – not a cause – of inflammatory processes [14]. The present case report lends support for Autier and colleagues’ idea [14]. The mechanism probably involves vitamin D-binding protein because inflammation and critical illness have been shown to coincide with lower levels of vitamin D-binding protein [15, 16]. We propose that lower vitamin D-binding protein underlies the lower 25(OH)D results of day 3. So far as we are aware, the present case report is the first longitudinal evidence of an acute, illness-related decrease in circulating 25(OH)D, which is superimposed onto the diurnal pattern of serum 25(OH)D.

Two recent publications report a consistent pattern of a diurnal rhythm in serum 25(OH)D across various population groups: Great Britain, China, Gambia, and India. Peak concentrations occur around midday, and nadir concentrations are from night into early morning [7, 8]. The report by Jones *et al.* showed that the diurnal rhythm is attributable to a parallel diurnal pattern in the levels of vitamin D-binding protein [7]. Vitamin D-binding protein is an acute phase reactant, with a half-life of less than 24 hours, and it has multiple functions that include actin scavenging, binding of fatty acids, chemotaxis, binding of endotoxins, influence on T cell response, and vitamin D-binding protein-macrophage activating factor [17].

The strengths of the present study include using multiple independently drawn samples taken at each time point to permit statistical comparisons across each day in one individual. Furthermore, the study design made possible the demonstration that there is within one individual a consistent pattern of blood 25(OH)D concentration, as shown on four independent study days over the span of 7 weeks. The diurnal phenomenon was not related to the timing of the daily dose of vitamin D, whether before or after blood sampling. We demonstrated the diurnal variation of 25(OH)D at a concentration range almost three times higher than what has been published previously.

The main limitation of this study is that it is a case report, and the potentially important decline in 25(OH)D concentrations in relation to illness needs to be confirmed with longitudinal measures of 25(OH)D involving many more participants. Testing at three time points on each day did allow us to identify a diurnal variation, but the limited number of sample times precludes knowing if we measured peak, trough, or intermediate concentrations.

## Conclusions

In conclusion, we demonstrated a repeatable and statistically significant diurnal pattern in blood 25(OH)D concentrations in one individual. We found no effect on blood 25(OH)D due to trough versus post-dose blood draws. An unexpected cold during the course of the study led to the observation that pre-symptomatic acute illness was related to acutely lower serum 25(OH)D levels. Because the daily, within-person variability in 25(OH)D is approximately 20%, it is clear that time of day for a blood draw contributes a systematic error in vitamin D nutritional assessment that is bigger than random analytical variability or choice of assay methodology.

## Additional file

Additional file 1: Repeated pattern of daily fluctuation in blood-spot 25(OH)D levels. Each dot on a line represents the mean measurement of five separately sampled blood-spot 25(OH)D test samples taken from one person. All individual test results are also represented. At days 1 and 2, the midday samples were taken 1 hour after the preceding daily, 5000 IU dose. At days 3 and 4, the midday samples were theoretical trough values, that is, sample taken approximately 24 hours after the preceding daily 5000 IU dose. *25(OH)D* 25-hydroxyvitamin D. (TIF 119 kb)

## References

[1] Barger-Lux MJ, Heaney RP. Effects of above average summer sun exposure on serum 25-hydroxyvitamin D and calcium absorption. J Clin Endocrinol Metab. 2002;87:4952–6. 10.1210/jc.2002-020636 12414856

[2] Armas LA, Hollis BW, Heaney RP. Vitamin D2 is much less effective than vitamin D3 in humans. J Clin Endocrinol Metab. 2004;89:5387–91. 10.1210/jc.2004-0360 15531486

[3] Sanders KM, Stuart AL, Williamson EJ, Simpson JA, Kotowicz MA, Young D, Nicholson GC. Annual high-dose oral vitamin D and falls and fractures in older women: a randomized controlled trial. JAMA. 2010;303:1815–22. 10.1001/jama.2010.594 20460620

[4] Ish-Shalom S, Segal E, Salganik T, Raz B, Bromberg IL, Vieth R. Comparison of daily, weekly, and monthly vitamin D3 in ethanol dosing protocols for two months in elderly hip fracture patients. J Clin Endocrinol Metab. 2008;93:3430–5. 10.1210/jc.2008-0241 18544622

[5] Binkley N, Carter GD. Toward Clarity in Clinical Vitamin D Status Assessment: 25(OH)D Assay Standardization. Endocrinol Metab Clin N Am. 2017;46:885–99. 10.1016/j.ecl.2017.07.012 29080641

[6] Sempos CT, Betz JM, Camara JE, Carter GD, Cavalier E, Clarke MW, Dowling KG, Durazo-Arvizu RA, Hoofnagle AN, Liu A, Phinney KW, Sarafin K, Wise SA, Coates PM. General Steps to Standardize the Laboratory Measurement of Serum Total 25-Hydroxyvitamin D. J AOAC Int. 2017;100:1230–3. 10.5740/jaoacint.17-0259 28766476

[7] Jones KS, Redmond J, Fulford AJ, Jarjou L, Zhou B, Prentice A, Schoenmakers I. Diurnal rhythms of vitamin D binding protein and total and free vitamin D metabolites. J Steroid Biochem Mol Biol. 2017;172:130–5. 10.1016/j.jsbmb.2017.07.015 PMC5571031 28732681

[8] Masood T, Kushwaha RS, Singh R, Sailwal S, Pandey H, Varma A, Singh RK, Cornelissen G. Circadian rhythm of serum 25 (OH) vitamin D, calcium and phosphorus levels in the treatment and management of type-2 diabetic patients. Drug Discov Ther. 2015;9:70–4. 10.5582/ddt.2015.01002 25788054

[9] Institute of Medicine Committee to Review Dietary Reference Intakes for Vitamin D, Calcium. The National Academies Collection: Reports funded by National Institutes of Health. In: Ross AC, Taylor CL, Yaktine AL, Del Valle HB, editors. Dietary Reference Intakes for Calcium and Vitamin D. Washington (DC): National Academies Press (US) National Academy of Sciences; 2011. 21796828

[10] Holick MF, Binkley NC, Bischoff-Ferrari HA, Gordon CM, Hanley DA, Heaney RP, Murad MH, Weaver CM. Evaluation, treatment, and prevention of vitamin D deficiency: an Endocrine Society clinical practice guideline. J Clin Endocrinol Metab. 2011;96:1911–30. 10.1210/jc.2011-0385 21646368

[11] Vieth R. Vitamin D supplementation, 25-hydroxyvitamin D concentrations, and safety. Am J Clin Nutr. 1999;69:842–56. 10.1093/ajcn/69.5.842 10232622

[12] Eyles D, Anderson C, Ko P, Jones A, Thomas A, Burne T, *et al.* A sensitive LC/MS/MS assay of 25OH vitamin D(3) and 25OH vitamin D(2) in dried blood spots. Clin Chim Acta. 2009;403(1–2):145–51. 10.1016/j.cca.2009.02.005 19232332

[13] ZRT Laboratory. Blood Spot Test Specifications 25-Hydroxy Vitamin D2/D3. http://www.zrtlab.com/media/1086/vitamin_d_bs_test_specs.pdf. Accessed 17 Aug 2018.

[14] Autier P, Boniol M, Pizot C, Mullie P. Vitamin D status and ill health: a systematic review. Lancet Diabetes Endocrinol. 2014;2:76–89. 10.1016/S2213-8587(13)70165-7 24622671

[15] Madden K, Feldman HA, Chun RF, Smith EM, Sullivan RM, Agan AA, Keisling SM, Panoskaltsis-Mortari A, Randolph AG. Critically Ill Children Have Low Vitamin D-Binding Protein, Influencing Bioavailability of Vitamin D. Ann Am Thorac Soc. 2015;12:1654–61. 10.1513/AnnalsATS.201503-160OC PMC5467088 26356094

[16] Ghafouri B, Carlsson A, Holmberg S, Thelin A, Tagesson C. Biomarkers of systemic inflammation in farmers with musculoskeletal disorders; a plasma proteomic study. BMC Musculoskelet Disord. 2016;17:206. 10.1186/s12891-016-1059-y PMC4862124 27160764

[17] Delanghe JR, Speeckaert R, Speeckaert MM. Behind the scenes of vitamin D binding protein: more than vitamin D binding. Best Pract Res Clin Endocrinol Metab. 2015;29:773–86. 10.1016/j.beem.2015.06.006 26522461

