# Supplementary figures and images for: 25-Hydroxyvitamin D variability within-person due to diurnal rhythm and illness: a case report
Source: J Med Case Rep. 2019 Feb 4;13:29. doi: 10.1186/s13256-018-1948-9 (PMC6360762; doi:10.1186/s13256-018-1948-9)

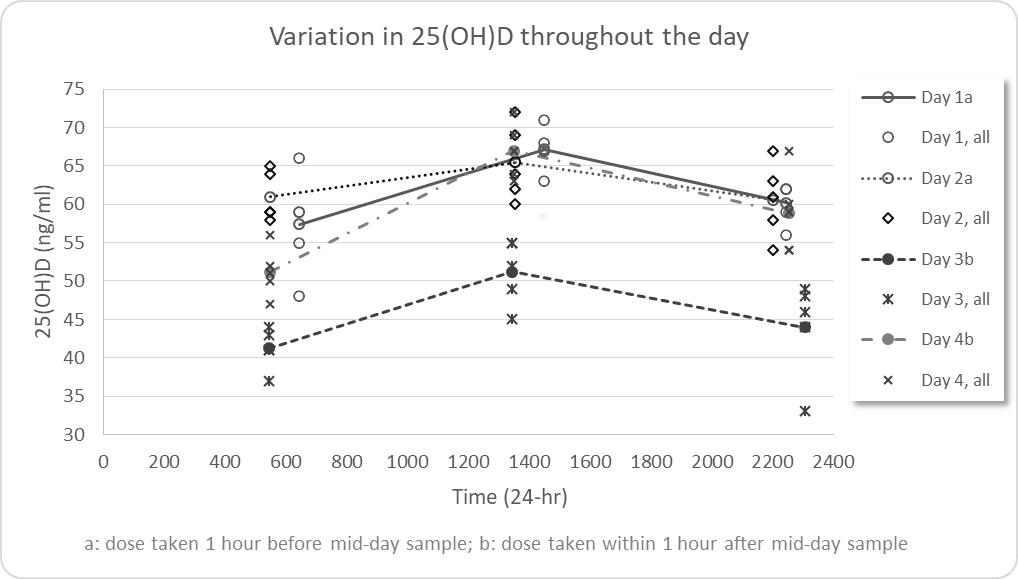

Supplement: Supplementary file 1 — Repeated pattern of daily fluctuation in blood-spot 25(OH)D levels. Each dot on a line represents the mean measurement of five separately sampled blood-spot 25(OH)D test samples taken from one person. All individual test results are also represented. At days 1 and 2, the midday samples were taken 1 hour after the preceding daily, 5000 IU dose. At days 3 and 4, the midday samples were theoretical trough values, that is, sample taken approximately 24 hours after the preceding daily 5000 IU dose. 25(OH)D 25-hydroxyvitamin D. (TIF 119 kb) [file 13256_2018_1948_MOESM1_ESM.tif]
